# Experiences with the Kono-S anastomosis in Crohn’s disease of the terminal ileum—a cohort study

**DOI:** 10.1007/s00423-020-01998-6

**Published:** 2020-10-06

**Authors:** K. Horisberger, D. L. Birrer, A. Rickenbacher, M. Turina

**Affiliations:** Department of Visceral and Transplantation Surgery, University Hospital of Zurich, Zurich, Switzerland

**Keywords:** Crohn’s disease, Terminal ileum, Ileocecal resection, Kono-S anastomosis

## Abstract

**Purpose:**

The most frequent long-term complication after ileocecal resection in Crohn’s disease is anastomotic recurrence and subsequent stenosis. Recurrence typically begins at the site of the anastomosis, raising the question of whether the surgical technique of the anastomosis could affect recurrence rates. Kono-S anastomosis is a hand-sewn antimesenteric functional end-to-end anastomosis that offers a wide lumen that is well accessible for endoscopic dilatation. The purpose of our study is to review the rate of postoperative complications almost 2 years after the introduction of this technique.

**Materials and methods:**

This is a prospective single-center cohort study of all consecutive patients with Crohn’s disease undergoing ileocecal resection. Patients’ characteristics as well as specific data for the surgical procedure and short-term outcome were evaluated.

**Results:**

Thirty patients were operated for Crohn’s disease of the terminal ileum (*n* = 24) or anastomotic recurrence (*n* = 6). Postoperative complications with a Clavien-Dindo Score ≥ IIIb were observed in three patients. One patient showed a hemorrhage and underwent surgical hemostasis. Two patients developed anastomotic leakage; in both cases, ileostomy was created after resection of the anastomosis. The median hospital stay was 9 days (IQR 7–12). A comparison with a historic group of conventionally operated patients of our hospital revealed no differences in short-term results except for the duration of surgery.

**Conclusion:**

The Kono-S anastomosis is associated with acceptable short-term results, complications, and recurrence rates comparable with the established anastomotic techniques. Longer operation times are observed, but the few published studies concerning long-term recurrence are promising.

## Introduction

Medical therapy has profoundly improved the course of Crohn’s disease (CD) and reduced the need for surgical interventions. While previous data describe such a need in about 74–80% of patients, this number was lowered by 30% after the introduction of infliximab [1–7]. After ileocecal resection, 50% of patients develop a recurrence, and reoperation rates gradually increase over time [8, 9].

This increase raises the question of whether alterations in the surgical technique may lead to lower reoperation rates. Whether radical resection of the mesentery affects reoperation rates in this way, as some authors have suggested, is currently being determined by a randomized controlled trial (NCT03769922) [10]. However, the most widely discussed technique in surgery is anastomosis. The efficacy and safety of the established types of anastomosis, such as end-to-end, side-to-end, and side-to-side, have been demonstrated. Comparison of stapled versus hand-sewn anastomosis with respect to the need for repeat surgery failed to show the superiority of one technique over the other [11, 12].

**Fig. 1:**
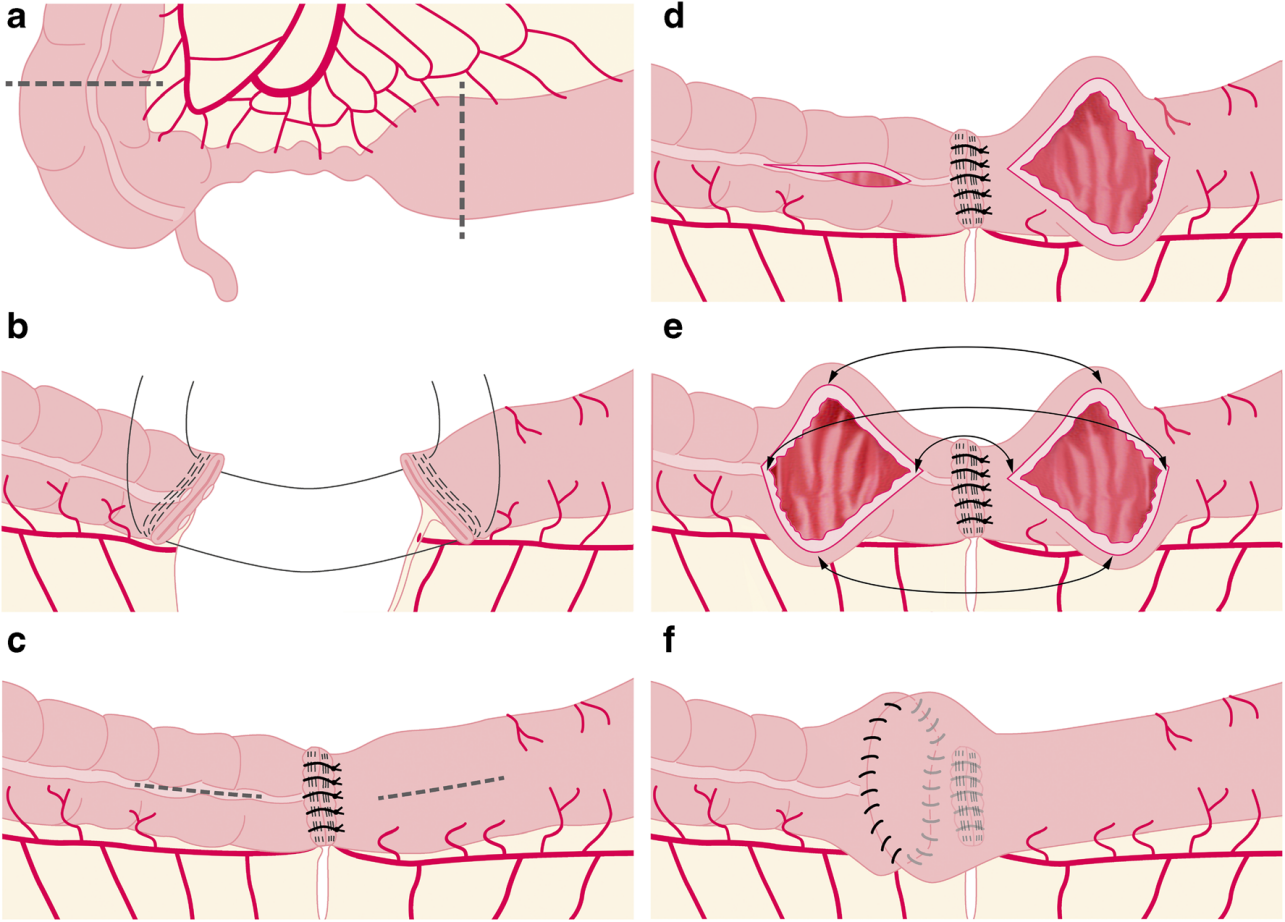
Surgical technique of Kono-S anastomosis. **a** The nearby mesentery of the ileocecal region that is to be excised is divided at the mesenteric wall of the bowel. The dotted line indicates the resection lines. **b** The intestine is transected by use of a linear staple cutter such that the mesentery is in the middle of the staple line and at a 90° angle to it. **c** Then, the staple lines are sutured together transversely to create a supporting column that is supposed to support the eventual dimension of the anastomosis. **d** Longitudinal enterotomies of 7 cm length are then performed at the antimesenteric aspect, beginning 1 cm from the supporting column. **e** The anastomosis is then created transversely in hand-sewn fashion. The backwall using a double-layer continuous manner, the front wall using a single-layered continuous suture. All sutures with 4/0 PDS. **f** The pale lines indicate the mesenteric side below the anastomosis with the supporting column that is created with the proximal and distal bowel stumps

In 2003, Kono established a new technique in order to reduce the rate of anastomotic strictures by recurrent CD (Fig. 1) [3]. Preliminary results showed an impressively low rate of recurrence requiring reoperation [13]. To date, there have been few comparative cohort studies with side-to-end and end-to-end anastomoses [14, 15]. In both comparisons, Kono-S anastomosis led to fewer short-term complications as well as improved long-term results with respect to anastomotic patency.

Here, we present the first cohort of all consecutive CD patients operated at a tertiary referral center for CD in our country. Our present study aimed to corroborate the results of earlier reports on the safety and efficacy of Kono-S anastomosis with short-term morbidity and rates of anastomotic leakage as the primary study endpoints.

## Patients and methods

### Patient selection

All consecutive patients with a diagnosis of CD undergoing ileocecal resection at our hospital from April 2018 to October 2019 were included in this cohort study. All patients eligible for ileocecal resection were offered the Kono-S anastomosis without any specific selection criteria. Three certified colorectal surgeons (K.H., A.R., and M.T.) performed the operations. During the study period, no patient underwent ileocecal resection with another type of anastomosis.

All patients received regular follow-up either at our hospital or by their treating gastroenterologist. All patients provided written, informed consent to the use of the Kono-S anastomosis as well as the analysis of their healthcare data. Institutional review board approval was provided (KEK-ZH-Nr. 2019-00208). The work has been reported in line with the STROCSS criteria [16].

### Control group

The control group was retrospectively extracted from our hospital database. All consecutive patients from March 2014 to December 2017 were analyzed. Patients with an emergency surgery were excluded as no certified colorectal surgeon performed the procedure.

### Follow-up

Most patients are regularly cared for and monitored in our endoscopy department. Internal follow-up consists of a regular postoperative visit at 3 and 6 months after surgery. In patients with external follow-up, we have requested documentation on clinical and endoscopic course and recurrence from the treating gastroenterologist.

### Surgical technique

Following intraoperative definition of appropriate resection margins, the transections of terminal ileum and colon are performed with a linear stapler perpendicular to the mesentery (Fig. 1). The center of the mesentery is thereby in the middle of the stapler line, and the mesentery is at a right angle to the stapler line. The opposing ends of the proximal and distal segments are then stitched together at the corners: the two stapler lines are sutured together using interrupted sutures (PDS 3–0 or 4–0). In case of a length mismatch of the stapler lines, the two sides are evenly distributed over the entire width.

The anastomosis is performed on the antimesenteric side of the bowel: the bowel is opened lengthwise at a distance of 1 cm from the stapler line, and the opening is then pulled in the lateral direction. The transverse opening needs to have a length of at least 7 cm. The posterior wall of the anastomosis is performed using a double-layer continuous suture and the anterior wall using a continuous single-layer suture (PDS 3–0 or 4–0). We did not measure the exact suturing time for the anastomosis, but we performed a comparison between the patient groups that each had exclusive ileocecal resection.

### Statistical analysis

Data are presented in a descriptive and a comparative statistical manner. Quantitative data are presented with median and interquartile range. Comparative data were analyzed with SPSS version 26 (SPSS Inc., Chicago, IL). Comparison of data was performed using chi-square test for categorical data and Mann-Whitney *U* test for nonparametric data. A *p* value of 0.05 was considered as statistically significant.

## Results

From April 2018 to October 2019, 30 patients were eligible for a reconstruction after ileocecal resection with a Kono-S anastomosis. Patient characteristics are shown in Table 1 and show no difference to the control group with standard anastomosis.

**Table 1:** Demographic and baseline characteristics of patients included

	Kono-S group *n* = 30	Control group *n* = 30	*p* value
Gender f/m (% female)	14/16 (46.7% f)	13/17 (43.3% f)	n.s.
Age (years [median; IQR])	32 [27–46.5]	33 [27.3–43.3]	n.s.
Primary resection/recurrence	24 (80%) / 6 (20%)	28 (93.3%)/2 (6.7%)	n.s.
BMI [median; IQR]	23 [19.7–27.7]	22.6 [19.7–25]	n.s.
Duration of disease (years [median; IQR])	9.5 [3–15.5]	8 [3–14.8]	n.s.
Perioperative medication			
Prednisone; dose [median; range]	11 (36.7%);	11 (36.7%)	n.s.
	10 mg [2.5–20]	20 mg [11–43]	
Budesonide	8 (26.7%)	10 (33.3%)	n.s.
Thiopurines	6 (20%)	3 (10%)	n.s.
Methotrexate	2 (6.7%)	0	n.s.
Biologics	19 (63.3%)	8 (26.7%)	0.008
Preoperative albumin level (g/l) [median IQR]	36 [30–38.5]	36.5 [31.8–40]	n.s.
Current smoker	8 (26.7%)	7 (23.3%)	n.s.
Preoperative interval between biologic medication and surgery	Days (median)	Days (median)	
All patients (*n* = 19 vs. *n* = 8)	36.5		n.a.
Infliximab (*n* = 6 vs. *n* = 2)	41.5	1 × 42; 1× unknown	n.a.
Vedolizumab (*n* = 4 vs. *n* = 2)	50	1 × 44; 1× unknown	n.a.
Adalimumab (*n* = 3 vs. *n* = 3)	13	22; 1× unknown	n.a.
Certolizumab (*n* = 2 vs. *n* = 1)	21	1 × 42	n.a.
Ustekinumab (*n* = 1 vs. *n* = 0)	42		

In all patients, the Kono-S technique was applied for an ileocolic anastomosis after either primary ileocecal resection or repeat resection of an ileocolic anastomosis. In 80% of patients, the resection was their first surgical treatment of abdominal CD. Median duration of the disease was 8 years. Five patients (16.7%) had a serum albumin level below 30 g/l. In another five cases (16.7%), preoperative albumin level was missing. In the control group, two patients underwent surgery for recurrent disease. Two patients of the control group showed a serum albumin level below 30 g/l, and in another twelve cases (40%), preoperative albumin level was missing.

In all but eight cases of each group, additional surgical steps were necessary (e.g., extensive adhesiolysis or intraoperative endoscopy; Table 2). In eight cases (26.7%) of the Kono-S group, an additional anastomosis or suture had to be performed for discontinuous Crohn’s enteritis or fistula to the sigmoid colon: additional anastomosis was not performed using the Kono-S technique. In one case, a fistula to the bladder was resected and closed. Six patients (20%), each of the Kono-S group of the control group, received a diverting loop ileostomy (Table 3).

**Table 2:** Procedural data

Intraoperative data	Kono-S group *n* = 30	Control group *n* = 30	*p* value
Laparoscopic resection	20 (66.7%)	14 (46.7%)	n.s.
Length of surgery (min [median; IQR])	256 [207.5–281.5]	206 [180–277]	n.s.
Length of surgery in exclusive IC resection (*n* = 8) (min [median; IQR])	192.5 [181–234]	155 [154–176]	0.015
Additional surgical intervention			
Adhesiolysis	5 (16.7%)	6 (20%)	n.s.
Stricturoplasty	2 (6.6%)	4 (13.3%)	n.s.
Diverting ileostomy formation	6 (20%)	5 (16.7%)	n.s.
Intra-abdominal abscess	5 (16.7%)	2 (6.7%)	n.s.
Enterocolic fistula resection	5 (16.7%)	8 (26.7%)	n.s.
Enterocutaneous fistula resection	1 (3.3%)	2 (6.7%)	n.s.
Perianal fistula surgery	1 (3.3%)	0	n.s.

**Table 3:** Indication for ileostomy

Indication for protective ileostomy	Kono-S (*n* = 6)	Control group (*n* = 6)
Serum albumin < 25 g/l	1	
Immunosuppression > 40 mg prednisone	2	1
Immunosuppression 20 mg + low serum albumin (< 30 g/l)	2	1
Simultaneous sigmoid/rectal resection + ileocecal resection	1	3
Extended adhesiolysis	–	1

In 20 (66.7%) patients of the Kono-S group and in 14 (46.7%) of the control group, the ileocolic resection was performed laparoscopically (*p* = 0.201). In ten patients (33.3%) of the Kono-s group, the resection had to be performed open, either due to interenteric abscess formation (*n* = 4), previous operations (*n* = 5), and, in one patient, pregnancy at the time of surgery (Table 2). One patient had to be converted to open surgery after diagnostic laparoscopy due to an extended abscess in the retroperitoneum. The Kono-S anastomosis was performed extracorporeally in all cases. The length of operation was a median of 256 min (IQR 207.5–281.5). In eight patients with exclusive laparoscopic IC resection (without additional procedures to other sites of the bowel), the length of operation was a median of 192.5 min (IQR 181–234) in the Kono-S group and 155 min (154–176) in the control group (*p* = 0.015).

### Postoperative complications

Eighteen patients of the Kono-S group showed minor complications: most of them had a Clavien-Dindo score of I or II: only one case of IIIa was observed (colonoscopy due to hemorrhage but without proof of bleeding). Three patients (10%) developed postoperative major complications ≥ Clavien-Dindo IIIb (Table 4). One patient suffered from a hemorrhage from a divided mesentery in acute Crohn’s inflammation and underwent reoperation to control the hemorrhage. Two patients suffered from anastomotic leakage (6.7%): both patients received surgery for recurrence of CD. In one patient with recurrent disease, we decided to forego a diverting ileostomy due to her concurrent pregnancy. However, she had already experienced a complicated course after initial ileocecal resection with anastomotic leakage. Five days after the creation of the Kono-S anastomosis, inflammatory parameters increased, and her clinical performance showed deterioration. The anastomosis showed a small leak, which was repaired using excision and suture of the defect, and a diverting loop ileostomy was performed. The other patient with a postoperative leak had previously undergone radical surgery for rectal cancer and near-total colectomy with a remaining of 20 cm of colon that was used as a colostomy. Due to recurrent CD, she had further undergone several segmental small bowel resections and had 120 cm of small bowel left. In order to preserve as much bowel as possible, the remaining 20 cm of colon was preserved, and a Kono-S anastomosis was created 20 cm before the colostomy. This patient had already shown an aggressive course of CD in her previous history with multiple enterocutaneous fistula. Ten days postoperatively, she developed peritonitis and showed a broad leakage of the suture and active colitis in the remaining colon. Completion colectomy with end ileostomy was created, after which she recovered uneventfully.

**Table 4:** Short-term outcomes

Postoperative data	Kono-S *n* = 30	Control group *n* = 30	*p* value
Minor complications (Clavien-Dindo < IIIb)	18 (62.1%)	21 (70%)	n.s.
Major complications (Clavien-Dindo ≥ IIIb)	3 (10%)	3 (10%)	n.s.
Reoperation	3 (10%)	3 (10%)	n.s.
Anastomotic leakage	2 (6.7%)	3 (10%)	n.s.
Hemorrhage with indication for surgical revision	2 (6.7%)		
Length of hospital stay (days [median; IQR])	9 [7–12]	9 [8–11]	n.s.

### Follow-up

Concerning the Kono-S group, regular follow-up at our hospital or by their treating gastroenterologist showed a median follow-up of 10 months (IQR 5.5–13). During this follow-up, 16 patients (53.3%) had undergone colonoscopy following a median 7 months (IQR 4–8.3) after surgery. In five of the remaining 14 patients, an ultrasound was performed, after a median interval of 6 months (IQR 5–11) to surgery. Altogether, follow-up by ultrasound or endoscopy was performed in 21 patients (70%). In three patients, endoscopic recurrence was found with Rutgeerts score 2. Medical treatment was initiated in these patients; there was no need for endoscopic or surgical treatment in the brief follow-up period. During follow-up, the ileostomy was closed in five of the six patients who received one after Kono-S anastomosis. Median time to reversal was 3 months (range 2–6 months).

The length of follow-up varies significantly between the Kono-S and the control group, as does the rate of recurrence (Table 5).

**Table 5:** Follow-up

	Kono-S *n* = 30	Control group *n* = 30	*p* value
Follow-up (months [median; IQR])	10 [5.5–13]	44 [26–55.8]	< 0.001
Endoscopic recurrence	3 (10%)	16 (53.3%)	0.013
Rutgeerts score of recurrences (i2–4; [median; IQR])	2 [2–2]	3 [2–4]	n.s.
Reoperation for recurrence	0	2 (6.7%)	n.s.

## Discussion

The most common site of CD involvement is the terminal ileum with a need for surgery in approximately half of cases [17, 18]. In many cases, the disease has advanced to such an extent that medical therapy can no longer offer relief, and surgery is the only way to achieve remission [18]. In fact, ileocecal resection has only recently been accepted as a choice of treatment in early as well as late stages [7]. Whatever the conditions for the initial ileocecal surgery, repeat resection due to symptomatic recurrence becomes necessary in roughly half of the affected patients [18–20]. This confronts surgeons with the question of whether the operative strategy and technique of their initial work could have been improved and thereby prevented the need for subsequent operations. [21]

Recurrence typically begins at the site of the anastomosis. According to the literature, about 80% of patients develop an endoscopic recurrence [3, 14]. Previous studies have shown that the extent of the surgery with respect to the length of the resected segment does not alter the rate of recurrence [11], shifting the attention to the technique of anastomosis formation in CD [3].

While a large randomized trial and a Cochrane meta-analysis showed no difference in recurrence rates after end-to-end versus side-to-side anastomosis [11, 12], a subsequent meta-analysis reported a significant advantage for side-to-side anastomosis [22]. The contrasting recommendations of the German guidelines (where all anastomotic techniques are considered to be equivalent) and the European Crohn’s and Colitis Organization (ECCO) guidelines (where a clear recommendation is given for a wide side-to-side anastomosis) mirror these conflicting results [23, 24]. In fact, only a few randomized controlled trials could be included in the meta-analysis, and evidence is still scarce [22, 25].

The more recent meta-analysis concludes that the larger side-to-side anastomosis is preventive of recurrence [22]. If increasing width of an anastomosis is preventive, the formation of a wide anastomosis should be the ultimate surgical purpose in resections for CD [26].

The Kono-S anastomosis offers an antimesenteric side-to-side suture technique that results in a large diameter, which should presumably help prevent anastomotic stenosis in recurrent CD [3]. In fact, all previous publications show impressively low symptomatic recurrence rates (Table 6) [14]. The first published randomized controlled trial by Luglio et al. demonstrated that endoscopic and clinical recurrence rates are significantly lower when using the Kono-S anastomosis compared with a standard stapled ileocolic side-to-side anastomosis [27]. On a meta-level, Alshantti et al. performed a systematic review and concluded that a significant reduction in endoscopic and surgical recurrence can be achieved using the Kono-S anastomosis [28]. However, it has to be noted that endoscopic recurrences are considerably more frequent than symptomatic recurrences [24]. An endoscopic recurrence is primarily treated with medication. However, fixed stenosis that may result from anastomotic recurrence is not accessible to medical treatment creating the need for endoscopic intervention or repeat surgery [29, 30]. This remains the case, despite the development of more potent medical therapies [7, 31]. The functional end-to-end configuration of the Kono-S anastomosis enables straightforward endoscopic access and withdrawal, providing a true benefit for endoscopists [32]. Endoscopic dilatation may be the first interventional treatment for stenosis of the anastomosis but is limited in angulated bowel [33]. However, information on angulation of anastomotic strictures is often lacking but may have important implications on efficacy [34]. Therefore, the accessibility and technical feasibility of a dilatation must be ensured. In addition, endoscopic dilatation is easier to perform after hand-sewn than after stapled anastomoses.

**Table 6:** Recurrence rates after Kono-S anastomosis

Author	Year	Number of patients	Asymptomatic	Recurrence			Follow-up (months)
				Clinical	Endoscopic	Surgical	
Fichera [19]	2012	46	Unknown	Unknown	11%	0	6.8
Katsuno [13]	2015	32	Unknown	Unknown	61.2%*	0	^a^
Kono [3]	2011	69	Unknown	Unknown	83%	0	^b^
Kono [14]	2016	144 Japan 29 USA	Unknown	Unknown Unknown	100% unknown	1.8% 0	65 (43–138) 32 (12–44)
Luglio [27]	2020	36	Unknown	8% at 12 mt 18% at 18 mt	22.2%	0	^c^
Seyfried [32]	2019	53	45.65%	nn	nn	0	12 (4–23)

^a^Katsuno: Follow-up for endoscopic recurrence: 14.8 months (3.-37); follow-up for surgical recurrence: 35 months (4–57)

^b^Kono: endoscopic recurrence: 83% at 1-year follow-up; 100% endoscopic recurrence at 5 years follow-up

^c^Luglio: Follow-up for endoscopic recurrence: 6 months; follow-up for surgical recurrence: 24 months

The Kono-S anastomosis combines the wide technique of an antimesenteric side-to-side hand-sewn suture with the straight accessibility of an end-to-end anastomosis [3]. In our Kono-S cohort, no symptomatic recurrence has occurred to date. Follow-up is short, and further prognosis of our cohort would be speculative. It is possible that over time, not only endoscopic recurrences will occur in the Kono-S group but also individual symptomatic surgical ones. In fact, the recurrence rate in the literature after Kono-S anastomoses is below 2% as shown in Table 6, while after classical surgical resection, symptomatic recurrence may occur in 30% of patients, and 5% have to undergo further surgical treatment within a year [31, 35]. In our series, the length of follow-up is significantly different between the Kono-S and the control group, so that the different rate of recurrence cannot be assessed conclusively, as it has to be assumed that more recurrences occur over time.

The perioperative results of our study provide a more critical assessment of anastomosis than other cohort studies [19, 32]. Patient selection in our hospital was not restrictive in any way. However, for patients at risk for anastomotic leakage, the large suture length of the anastomosis may also imply an increased risk of subsequent leakage. In fact, the leakage rate of 6.7% in our cohort, which is in line with most studies on ileocolic anastomoses, may not allow a firm conclusion due to the small number of patients. In other cohorts, the leakage rate in patients with Kono-S anastomosis is promisingly low according to the few currently available studies.

There are some limitations of our study. First, the study was not designed to be randomized from the beginning, and therefore, the comparison with the historical group may be biased by factors other than the type of anastomosis. Secondly, the follow-up is short, and therefore, no final assessment of the recurrence rate can be made. A further weakness of our study is that we did not measure the time for anastomosis. In fact, this factor may hinder some to adopt this new technique. For better understanding, we have listed additional operations that are performed in Table 2, as these also prolong the operating time and have separately listed the average surgery time of patients who had only undergone ileocecal resection. Although a shorter operation time for the exclusive IC resection is shown and although we did not specifically measure the exact time needed to complete the anastomosis, it was felt to significantly lengthen the operation. The comparison with the formerly performed stapled side-to-side anastomosis at our department correspondingly shows a significant longer operation time for Kono-S. There are no published data on the length of surgery for Kono-S anastomosis compared with other techniques, but the manifestly longer time needed may indeed be of concern for institutions with tight elective schedules.

## Conclusion

Firm recommendations as to whether the Kono-S anastomosis should become the technique of choice following ileocolic resection for CD would currently be premature, although the first recently published randomized control trial supports its use in such a context [27]. The few published reports, including ours, highlight the potential advantages of this new technique but also underline the need for further research and evidence [36].

## Data Availability

Due to the sensitive nature of the data in this study, raw data would remain confidential and would not be shared.
